# The Power of Words: Appearance Comments from One’s Partner Can Affect Men’s Body Image and Women’s Couple Relationship

**DOI:** 10.3390/ijerph18179319

**Published:** 2021-09-03

**Authors:** Elena Fornaini, Camilla Matera, Amanda Nerini, Giulia Rosa Policardo, Cristian Di Gesto

**Affiliations:** 1 School of Psychology, University of Florence, 50137 Florence, Italy; elena.fornaini@stud.unifi.it; 2 Department of Education, Languages, Intercultures, Literatures and Psychology, University of Florence, 50135 Florence, Italy; nerini@psico.unifi.it; 3 Department of Health Sciences, University of Florence, 50139 Florence, Italy; giuliarosa.policardo@unifi.it (G.R.P.); cristian.digesto@unifi.it (C.D.G.)

**Keywords:** appearance commentary, comments, partner, body dissatisfaction, body compassion, couple satisfaction, relationship quality

## Abstract

Background: The purpose of the present study was to examine, through an experimental vignette design, the effects of appearance-related comments from one’s partner on body image and the perceived quality of one’s relationship. Body image was considered both in negative (body dissatisfaction) and positive (body compassion) terms. Methods: Appearance-related commentary from one’s partner was manipulated through a vignette describing the purchase of a swimsuit. The participants (*n* = 211) were women and men who were randomly assigned to one of the three experimental conditions (positive comment, negative comment, no comment). Results: A series of ANOVAs showed different findings for women and men. Being criticized for body weight and shape caused an increase in body dissatisfaction and a decrease in body compassion in men but not in women. Regarding couple satisfaction, women who imagined receiving a compliment about their body perceived being more accepted by their partner and were less afraid of being abandoned or rejected. Conclusions: Our findings highlight the importance of appearance-based comments from one’s partner on men’s body image and on women’s perception of their couple relationship. Therefore, appearance comments might be addressed by interventions aimed at enhancing positive body image, reducing body dissatisfaction, and fostering couple relationships, carefully considering sex differences.

## 1. Introduction

Societal ideals of beauty and expectations about appearance are commonly conveyed through different sociocultural channels [1,2]. A large amount of literature has shown that interpersonal experiences and interactions can express, model, or reinforce cultural standards of beauty [1]. For instance, appearance-related commentary from peers and parents are pivotal in the development and maintenance of body image and eating concerns [3,4,5,6]. Less explored is the influence of romantic partners, who can have a positive impact on both the physical health and psychological well-being of individuals [7]. Examining appearance-related commentary from one’s partner is quite important if we think about the role of appearance and reciprocal appreciation within a romantic relationship. The present study contributes to the literature on this issue by examining, through an experimental vignette design, the effects of appearance-related comments from one’s partner on body image and the couple satisfaction of both women and men.

### 1.1. Appearance-Related Commentary and Body Image

The detrimental effect of negative appearance-related commentary and teasing on body dissatisfaction is well documented. Negative comments may reinforce sociocultural standards of beauty by promoting their internalization [8,9,10,11]. Given that the thin ideal for women and the mesomorphic ideal for men are almost unattainable, the internalization of these ideal standards may contribute to the development of poor body image and eating disturbance [2,12].

Some studies have focused on the role of positive appearance-related commentary, producing discordant results. Some research has suggested that positive comments as well as criticisms contribute to body dissatisfaction because both of these types of comments may trigger self-objectification [13,14,15]. Differently, other studies have suggested that compliments about appearance can have beneficial effects, such as enhancing one’s self-esteem and self-confidence [16,17,18].

To date, only a few studies have examined the effects of comments received from one’s romantic partner, showing that criticism about appearance from one’s partner is associated with body dissatisfaction, body image concerns [19,20,21], shame and self-doubt, self-consciousness, and insecurity about appearance [22]. Positive comments about weight and shape are associated with decreased body dissatisfaction [19,23], less eating concerns [19], and lower anxiety related to body image [21]. Most of these studies have focused on the negative factors associated with body image. An exception is represented by a study conducted by Goldsmith and Byers (2016) [22], showing that compliments from one’s romantic partner are associated with higher confidence and acceptance of one’s body. These findings are quite interesting if we consider that positive body image can help people appreciate, respect, and celebrate their body with favorable outcomes on their overall well-being [24,25].

One component of positive body image is body compassion, which is a fresh construct that is largely informed by self-compassion theory [26]. Self-compassion involves being open to one’s suffering through a self-kindness attitude and a nonjudgmental understanding of one’s pain and failures by seeing one’s own experience as a part of the common human experience [26]. Recent systematic reviews have shown that self-compassion is a protective factor against poor body image and eating pathology [27,28]. In body compassion’s conceptualization, the three components of self-compassion (self-kindness, common humanity, and mindfulness) are applied with a shift in emphasis from the “self” to the “physical self”; the concept of multidimensional body image (appearance, health/illness, competence/fitness) [29] is incorporated as well [30]. In other words, body compassion is described as a feeling of compassion directed towards one’s body. Emerging research suggests that body compassion is a protective factor against body and eating difficulties [31]. In addition, body compassion seems to buffer the effect of major life events (experiences of an abrupt life change, which may generate serious and long-lasting effects) on binge eating among women [32]. To the best of our knowledge, no previous research has investigated the relationship between appearance-related commentary and body compassion. In our study we considered body image not only in a negative way, but also in positive terms, which may improve our understanding of the influence of commentary on appearance from one’s partner as either protective or risk factors.

### 1.2. Appearance-Related Commentary and Relationship Satisfaction

Some studies have also investigated the effect of appearance-related commentary from one’s partner on couple satisfaction among heterosexuals couples. Indeed, some literature has shown that body satisfaction and couple satisfaction are interrelated [33,34,35,36].

Interestingly, positive comments about appearance from romantic partners are associated with both relationship [19,23] and sexual satisfaction [23]. Receiving compliments and reassurance about one’s weight can be beneficial for psychological well-being and the perceived quality of one’s couple relationship [37,38].

The impact of negative comments on relationship satisfaction is less clear. Sheets and Ajmere (2005) [39] found that criticism was associated with lower relationship satisfaction. College students who took part in their study were asked whether they or their partner had suggested the idea of losing weight to one another. Women who were told to lose weight and men who were told to gain weight reported less relationship satisfaction than participants who had not received this kind of comments; women who were told to gain weight did not report decreased couple satisfaction compared to those who were told to lose weight. The authors suggest that people expect their romantic partners to act as a protective factor against social standards of beauty; if one’s partner reinforces these ideals, relationship satisfaction might decrease [39]. A significant relationship between negative comments and couple satisfaction was not found in other studies [19,23].

At present, only one experimental study [40] has investigated responses to appearance feedback from one’s romantic partner, so inference concerning the causal relationship between comments and body dissatisfaction cannot easily be drawn. Brown and colleagues (2013) [40] examined the affective and cognitive reactions to women to imagined feedback from their partner by using a vignette through which they manipulated enhancing, verifying, or devaluing feedback. According to their findings, women who were more dissatisfied with their appearance felt happier but less understood when they received enhancing feedback, while they felt more upset but believed that they were perceived more accurately after verifying feedback [40]. Thanks to its experimental design, this study provided evidence concerning the causal effects that comments from one’s partner can have on the affective and cognitive reactions of women. However, the authors did not examine the effects of negative and positive comments on either the participants’ body image or relationship satisfaction, which was instead the focus of our study.

### 1.3. The Present Study

Although the above studies were promising in showing a significant association between appearance-related commentary from one’s partner and both body image and relationship satisfaction, they suffer from several limitations. First of all, body image has almost always been considered in terms of body dissatisfaction, rather than in terms of positive aspects. Secondly, correlational evidence has been mostly provided on this issue. Finally, a great deal of research has focused on women, almost ignoring how men’s body image and relationship satisfaction can be related to their partners’ commentary.

The present study aimed to fill these gaps by examining, through an experimental vignette design, the effect of appearance-related commentary from one’s partner on both body image and relationship satisfaction in men and women with a couple relationship. Notably, body image was considered both in negative (body dissatisfaction) and positive (body compassion) terms. We tested the following hypotheses:

Participants exposed to a positive comment about their physical appearance would report less body dissatisfaction and greater body compassion than those who were exposed to a negative comment and those who received no comment (Hypothesis 1). Participants exposed to a negative comment would report greater body dissatisfaction and less body compassion compared to those who were exposed to a positive comment and those who received no comment (Hypothesis 2). Respondents who were exposed to a positive comment would report a better perceived quality of their couple relationship than participants in the negative comment and no comment conditions (Hypothesis 3).

## 2. Materials and Methods

### 2.1. Research Design

We adopted an experimental, between-subject design in which the independent variable was the partner’s commentary (positive, negative, control), which was manipulated by presenting participants with a vignette. The assignment to the various experimental conditions occurred randomly, but separately for women and men, in order to ensure that an overall similar number of male and female participants were distributed among the different experimental conditions. The numbers of men and women in each group are reported in Table 1 and Table 2.

### 2.2. Participants

The participants were 211 people (101 men and 110 women) living in Italy. A power analysis using G*Power [41] indicated that a sample of 211 was sufficient to detect medium effects (effect size = 0.25) with 80% power using an ANOVA with six groups (3 × 2) and with alpha set at 0.05. We can then consider our sample adequate for testing our hypotheses.

The criterion for inclusion was to have been involved in a heterosexual romantic relationship for at least one year.

The female participants were aged between 19 and 72 (*M* = 31.88, *SD* = 12.87), and their average Body Mass Index (BMI) was 21.58 (*SD* = 3.04, ranging from 16.02 to 35.43). Their mean relationship length was around ten years (*M* = 10.37, *SD* = 11.51). The majority of women were of Italian nationality (97.3%), and most of them lived in central Italy (95.5%). Regarding civil status, 64.5% were unmarried, and 35.5% were married/cohabitating. Regarding education, 56.4% had completed high school, 31.8% had master’s degrees, 7.3% had bachelor’s degrees, 3.6% had completed secondary school, and 0.9% reported a different degree (i.e., specialization course). With respect to occupation, 51.9% were students, 23.6% had full-time employment, 10.9% had part-time employment, 5.5% had occasional work, 4.5% reported they were looking for their first job, 1.8% were retired, and 1.8% were unemployed.

The male participants were aged between 20 and 74 (*M* = 35.75, *SD* = 19.95), and their average BMI was 24.25 (*SD* = 3.28, ranging from 17.64 to 33.22). Their mean relationship length was around twelve years (*M* = 12.10, *SD* = 12.22). All of the men were of Italian nationality, and most of them lived in central Italy (96%). Regarding their civil status, 51.5% were unmarried, 46.5% were married/cohabitating, and 2% were separated/divorced. Regarding education, 47.5% had high school diplomas, 22.77% had bachelor’s degree, 21.8% had master’s degrees, 6.9% had completed secondary school, and 1% reported a different degree (i.e., Ph.D.). Half of them (50.5%) had full-time employment, 37.6% were students, 5.9% had part-time employment, 3% were retired, 1% had occasional work, 1% reported that they were looking for their first job, and 1% was unemployed.

### 2.3. Measures

In the present study, appearance-related commentary from one’s partner was manipulated through a vignette describing the purchase of a swimsuit (bikini for women and speedos for men) along with one’s partner, during which body image is likely to be especially salient. Previous studies showed that for many women, wearing a swimsuit is the most uncomfortable body-related situation [42] and shopping for or trying on bathing suit results in a distressing experience for them [43] that can trigger self-objectification [44]. Additionally, men who were asked to try on speedos (self-objectification situation) reported negative outcomes in terms of self-esteem and body shame compared to those who were asked to wear a sweater [45]. A study conducted on a large sample showed that 16% of men and 31% of women felt so uncomfortable with their appearance that they avoided wearing swimsuits in public [46].

In the present study, the respondents were asked to imagine trying the swimsuit on and, once wearing it, to pull back the curtain in order to hear their partner’s opinion on how they looked (see Appendix A for the complete vignettes). The vignette went on, reporting that the partner expressed a comment, which was different according to the experimental conditions: in the positive comment condition, the partner expressed some compliments, while in the negative comment condition, the partner expressed some criticism about the partners’ body weight and shape. In the control condition, no vignette was presented to the participants.

The participants were asked to read the vignette carefully and calmly, imagining themselves in the described situation. We preferred not to use a pre-test assessing body image in order to avoid any influence on participants’ perception of the situation described in the vignette. According to Pasnak (2018) [47], the use of pretests could decrease the internal validity of experiments (i.e., render them incapable of completely proving what they are intended to prove). After the presentation of the vignette, the participants completed items and scales aimed to assess the following variables.

*Manipulation check:* An item asking the participants to judge the comment they had received constituted our manipulation check. Participants provided their responses on a 9-point Likert scale from (1 = completely negative; 9 = completely positive). Participants in the no comment condition did not complete this measure.

*Body dissatisfaction:* Female body dissatisfaction was measured through the Italian version [48] of the *Body Shape Questionnaire-14* (BSQ-14) [49], which is composed of 14 items (e.g., “Has seeing your reflection—e.g., in a mirror or shop window—made you feel bad about your shape?”) with a 6-point Likert-type response format (1 = never; 6 = always). Higher scores indicate higher levels of body dissatisfaction (*α* = 0.90).

Male body dissatisfaction was measured through the Italian version [50] of the *Male Body Attitudes Scale* (MBAS) [51], which is composed of 24 items with a 6-point Likert-type response format (1 = never; 6 = always). The MBAS is composed of three subscales: *Muscularity* (10 items, e.g., “I think my chest should be larger and more defined”) to assess men’s attitudes toward their muscularity, *Low Body Fat* (8 items, e.g., “I think I have too much fat on my body”) to measure men’s attitudes toward their body fat, and *Height* (2 items, e.g., “I wish I were taller”) to assess men’s attitudes toward their height. Higher scores indicate higher levels of body dissatisfaction. In the present study, the Cronbach’s *α* for the subscales were the following: *Muscularity* = 0.85; *Low Body Fat* = 0.92; *Height* = 0.85; for the MBAS total, it was 0.83.

*Body compassion:* Body compassion was measured through the Italian version [52] of the *Body Compassion Scale* (BCS) [30], which is composed of 23 items with a 5-point Likert-type response format (1 = almost never; 5 = almost always). The Italian version of the BCS proved to be valid and reliable with both women [52] and men [53], for whom the factor structure of the scale was exactly the same. The BCS is composed of three subscales: *Defusion* (9 items, e.g., “When I am feeling physically uncomfortable, I tend to obsess and fixate on everything that is wrong”) to assess an attitude of decentering rather than emphasizing over-identification with one’s body inadequacies and failure; *Common Humanity* (9 items, e.g., “When I feel my body is inadequate in some way, I try to remind myself that feelings of inadequacy are shared by most people”) to measure the recognition of interconnectedness between people given by life experience through the physical interface of the body; and *Acceptance* (5 items, e.g., “I am tolerant of my body’s flaws and inadequacies”) to assess non-judgmental acceptance of appearance, state of health, and the function of one’s body as it is in the present. In the present study, the Cronbach’s *α* for the subscales were the following: for women, *Defusion* = 0.87; *Common Humanity* = 0.93; *Acceptance* = 0.93; BCS total score = 0.82; for men, *Defusion* = 0.88; *Common Humanity* = 0.94; *Acceptance* = 0.91; BCS total score = 0.85.

*Quality of couple relationship*: Relationship affectivity was measured through five subscales of the *Couple’s Affectivity Scale* (CAS) [54]. The CAS is a multidimensional measure aimed at examining several aspects of a couple’s relationship. The CAS was developed and validated in the Italian context with both women and men. Items have a 5-point Likert-type response format (1 = never; 5 = always). In the present study, we used the following subscales: *Self-disclosure* (SD; 4 items, e.g., “You have expressed to your partner unpleasant events that occurred during the day”) to measure willingness and openness to dialogue with a partner; *Partner-disclosure* (PD; 5 items, e.g., “Your partner has told you about his feelings”) to assess the respondents’ perception of their partner’s openness to expressing ideas and feelings; *Perceived Partner Responsiveness* (PPR; 5 items, e.g., “Your partner has shown that he esteems you”) to measure the respondents’ perception of the ability of their partner to express understanding, affection, esteem, and support; *Relational Communication* (RC; 3 items, e.g., “You have talked with your partner about your relationship”) to assess the ability to talk about the relationship and address couple problems; and *Fears of being Abandoned and Rejected* (FAR; 3 items, e.g., “You have happened to be afraid of loneliness”) to measure the fear of being abandoned and not accepted by the partner. For male participants, in the present study, the Cronbach’s *α* of the scales were acceptable, except for the FAR subscale: SD = 0.76; PD = 0.67; PPR = 0.82; RC = 0.74; FAR = 0.42. Due to its scarce reliability, FAR was not used for men in our analyses. For the female participants, the Cronbach’s *α* of the scales was acceptable, except for the RC subscale: SD = 0.76; PD = 0.73; PPR = 0.84; RC = 0.52; FAR = 0.60. Due to its scarce reliability, RC was not used for women in our analyses.

Each participant reported age, sex, the length of their relationship (expressed in months), educational level, and occupational status. We calculated BMIs (kg/m^2^) using the participants’ reported weights and heights.

### 2.4. Procedure

The study procedure was approved by the Ethical Committee of the university with which the authors are affiliated. Using opportunistic sampling techniques, we recruited the study participants from the School of Psychology at the university with which the authors were affiliated, who were asked to involve other participants among their networks. We asked the participants to take part in a study on body image. Participation in the study was voluntary, and we did not provide incentives to the participants. The participants provided their informed consent prior to completing the questionnaire, and their responses were recorded anonymously in full compliance with the privacy regulations. On average, each participant took 25 min to complete the questionnaire. The participants were randomly assigned to one of the three experimental conditions (positive comment condition, negative comment condition, control condition). After completing the questionnaire, each respondent was carefully debriefed.

### 2.5. Data Analysis

Descriptive statistics were calculated for all of the variables. We then performed a series of Univariate Analyses of Variance (ANOVAs) in order to test if the participants differed for key variables potentially associated with body image or relationship satisfaction (BMI, age, relationship length) based on either the experimental condition to which they were assigned or on their sex. A main effect of condition did not emerge for any of the variables (BMI, *F*_(2,205)_ = 1.00, *p* = 0.37; age, *F*_(2,205)_ = 0.31, *p* = 0.73; relationship length, *F*_(2,205)_ = 0.49, *p* = 0.61), but significant differences were found with respect to sex for BMI (*F*_(2,205)_ = 38.38, *p* < 0.001) and age (*F*_(2,205)_ = 4.69, *p* < 0.05) (relationship length, *F*_(2,205)_ = 1.08, *p* = 0.30). Given these sex differences, we decided to analyze the data for women and men separately. We thus conducted a series of ANOVAs including the experimental condition as the independent variable, while body dissatisfaction, body compassion, and quality of couple relationship were the dependent variables.

## 3. Results

An ANOVA on our manipulation check revealed a main effect of the experimental condition for both women, *F*_(1,72)_ = 107.52, *p* < 0.001, *η²* = 0.60, and men, *F*_(1,66)_ = 18.84, *p* < 0.001, *η²* = 0.22. The women in the negative comment condition reported a more negative judgment from the comment (*M* = 2.53) than those in the positive comment condition (*M* = 7.25). Analogously, the men in the negative comment condition reported a more negative judgment from the comment (*M* = 4.91) than those in the positive comment condition (*M* = 7.06).

A series of univariate ANOVAs were performed separately on the dependent variables for women and men. The respective means are displayed in Table 1 and Table 2.

The ANOVA revealed no significant effect of the experimental condition of the women’s body dissatisfaction, *F*_(2,101)_ = 0.31, *p* = 0.73. With regard to the men’s body dissatisfaction, the ANOVA revealed a significant effect of the experimental condition on *Low body fat*, *F*_(2,95)_ = 4.06, *p* < 0.05, *η²* = 0.08 and on total MBAS, *F*_(2,98)_ = 3.16, *p* < 0.05, *η²* = 0.07. In particular, post hoc analyses with Bonferroni correction showed that participants who received the negative comment reported greater body dissatisfaction with body fat (*M* = 3.41) than those who received the positive comment (*M* = 2.60) (*p* < 0.05); no significant differences emerged between either the positive or the negative comment condition and the control one (*M* = 2.79). Similarly, men in the negative comment condition reported greater body dissatisfaction (*M* = 2.95) than those in the positive comment (*M* = 2.59) and control conditions (*M* = 2.57); nonetheless, post hoc analyses did not reveal significant differences among the three groups. No significant effect of the experimental condition emerged on the *Muscularity*, and *Height* subscales (*F*_(2,94)_ = 0.16, *p* = 0.86, and *F*_(2,98)_ = 1.89, *p* = 0.16, respectively).

For women, the ANOVA revealed no significant effect of the experimental condition on body compassion (BCS total score, *F*_(2,103)_ = 0.94, *p* = 0.39; *Defusion*, *F*_(2,106)_ = 0.75, *p* = 0.47, *Common Humanity*, *F*_(2,106)_ = 0.66, *p* = 0.52; *Acceptance*, *F*_(2,106)_ = 1.68, *p* = 0.19). For men, a significant effect of the experimental condition was found on the total BCS score *F*_(2,97)_ = 3.05, *p* = 0.05, *η²* = 0.06. Post hoc analyses with Bonferroni correction showed that the participants in the control condition (*M* = 3.36) reported greater acceptance of their body than those who received the negative comments (*M* = 3.10) (*p* = 0.05); body compassion was not different in the participants who received the positive comments (*M* = 3.29) and the participants in the other conditions. No significant effect of the experimental condition emerged on the BCS subscales (*Defusion*, *F*_(2,98)_ = 1.88, *p* = 0.16; *Common Humanity*, *F*_(2,98)_ = 0.19, *p* = 0.83; *Acceptance*, *F*_(2,97)_ = 1.73, *p* = 0.18).

With regard to the women’s quality of couple relationship, the ANOVA revealed a main effect of the experimental condition on FAR, *F*_(2,107)_ = 3.93, *p* < 0.05, *η²* = 0.07. Post hoc analyses with Bonferroni correction showed that women who received positive comments reported lower levels of fear of being abandoned or rejected by their partner (*M* = 0.57) than those who received negative comments (*M* = 1.03) (*p* < 0.05). Differences between either the positive or negative conditions and the control one (*M* = 1.00) were not statistically significant. No significant differences emerged with respect to the experimental condition on SD, *F*_(2,107)_ = 0.01, *p* = 0.99, PD, *F*_(2,106)_ = 0.27, *p* = 0.77, and PPR, *F*_(2,105)_ = 0.77, *p* = 0.46.

For men, there was hardly any effect of the experimental condition on the quality of their couple relationship: SD, *F*_(2,96)_ = 0.11, *p* = 0.89; PD, *F*_(2,96)_ = 0.25, *p* = 0.78; PPR, *F*_(2,96)_ = 0.48, *p* = 0.62, and RC, *F*_(2,95)_ = 0.54, *p* = 0.59.

## 4. Discussion

The aim of the current research was to examine, through an experimental vignette design, the effect of appearance-related commentary from one’s partner on body image and relationship satisfaction in women and men. Comment manipulation through the vignette was shown to be effective; however, the effect size of the experimental condition was stronger for women than for men. We hypothesized that the participants exposed to a positive comment about their physical appearance would report less body dissatisfaction and greater body compassion than those who were exposed to a negative comment or who received no comment (Hypothesis 1). Participants exposed to a negative comment were hypothesized to report greater body dissatisfaction and less body compassion compared to those who were exposed to a positive comment or who received no comment (Hypothesis 2). Respondents exposed to a positive comment were predicted to report a better perceived quality of their couple relationship than participants in the negative comment and no comment conditions (Hypothesis 3).

Contrary to our expectations, the comment did not have a significant effect on the women’s body image that could be conceptualized in either positive or negative terms. These results are in contrast to correlational studies that highlighted a significant association between appearance-related commentary and women’s body dissatisfaction [15,16,17], especially with regard to one’s partner’s comments [19,20,22]. The comments did not affect the women’s body compassion either. This finding may suggest that receiving comments related to appearance from their partner does not affect women’s body image. Nevertheless, the lack of a significant effect of comments on women’s body image could be due to the fact that women just imagined receiving a comment from their partner, but they did not actually receive it. They might not perceive the comment as sufficiently realistic, or they might not identify enough with the depicted situation. Unfortunately, we did not include checks that could control for these kinds of perceptions in our study.

Notably, a significant effect of the comment on body image emerged among men, which confirmed previous correlational evidence [22,23]. Men receiving a negative comment reported greater levels of body dissatisfaction with body fat than those receiving a positive comment. The comment did not affect the men’s dissatisfaction with their height and muscularity. This result was consistent with the type of comment that the men imagined receiving, which focused on body weight and shape, rather than on other physical features such as height or muscularity.

Interestingly, the men showed also significant results concerning the effect of appearance-related commentary on body image in positive terms (body compassion). Participants who imagined receiving negative comments reported lower body compassion than those receiving no comment. This finding seems to suggest that being criticized by one’s partner may discourage an attitude of tolerance and non-judgment towards the flaws and inadequacies of one’s body. Nevertheless, differences among the three conditions did not emerge for the different body compassion subscales.

With respect to couple satisfaction, some interesting results emerged for women. The women receiving t positive comments reported lower levels of fear of being abandoned or rejected by their partner than those receiving the negative comment. Therefore, positive comments on body weight and shape seemed to play a protective role with respect to the fear of being abandoned and not accepted by the partner, which negatively influenced couple satisfaction [54]. These findings were consistent with correlational studies that have highlighted how compliments can be related to the perceived quality of a couple relationship and dyadic satisfaction [19,20,38]. Receiving compliments and reassurances about weight and appearance from one’s partner can help people develop a more accurate perceived partner satisfaction with their body, which has been proven to be a mediator for the association between body satisfaction and relationship satisfaction [34]. The received comments did not have a significant impact on the quality of couple relationship among men.

In summary, our findings only seem to confirm the role of appearance-related commentary by one’s partner in male body image; in particular, negative comments showed a detrimental effect on the men’s dissatisfaction with their bodies and threatened their body compassion, with possible further negative outcomes [55,56]. Similar findings were not obtained with women, for whom one’s partner’s comments concerning appearance affected some aspects of their relationship satisfaction, rather than their body image. For women, the positive comments played a protective role against the fear of being abandoned and rejected by their partner, thus promoting relational well-being. The findings of the present study are quite important, as this is the first time that a cause–effect relationship between a partner’s positive comments and the perceived quality of a couple relationship has been found.

Although this study contributes to the literature as one of the first attempts to experimentally investigate the effects of appearance-related comments from one’s partner on body image and relationship satisfaction in men and women, the present findings should be interpreted in the context of certain limitations. First, the comments were not directly received from one’s partner, but was instead transmitted through a vignette. It is possible that some participants (e.g., older women) were not able to imagine themselves in the described situation or did not perceive the comment as sufficiently realistic. Future studies might use further manipulation checks or different kinds of scenarios to allow as many people as possible to identify with the situation; an upper age limit on the sample could be established as well, given that older women might be less likely to receive appearance-related comments in their real life. Moreover, receiving a comment in a highly controlled setting may be different from receiving it in everyday life; therefore, these findings should be generalized with caution. Second, although the random assignment of participants to the experimental conditions should ensure an equal distribution, we did not use a pre-test. As a consequence, we were not able to check for homogeneity in the three groups before the vignette was presented. Third, given that significant differences between women and men were found with respect to BMI and age, we had to perform our analyses on the two groups separately, which slightly decreased the power of the study. Therefore, it would be necessary to replicate our findings with a larger sample of female and male participants. Fourth, weight and stature data reported by participants were used to calculate their BMIs, so this information might be biased; fortunately, BMI was not a key variable in our study. Finally, this research was conducted on heterosexual participants; the study could be replicated with homosexual participants to check for any differences based on sexual orientation. This would be interesting considering that a large amount of literature has shown that homosexual men report greater body dissatisfaction than heterosexual men [57]. Future research could also experimentally investigate reciprocal influences between partners. This might be possible by recruiting both members of the couple and assigning them to the same experimental condition.

## 5. Conclusions

The results of the present study seem to suggest that future research could investigate the effect of appearance-related commentary from significant others on both body compassion and other positive characteristics of body image, such as body appreciation and broadly conceptualizing beauty. It would also be interesting to investigate the effect of one’s partner’s comments about appearance on sexual satisfaction, which seems to act as a mediator in the relationship between body satisfaction and relationship satisfaction [22,33,34,35].

In regard to practical implications, our findings suggest that one’s partner may be involved in interventions aimed at promoting body and couple satisfaction. It is also necessary to consider the population of men, who are traditionally considered to be less at risk regarding to body dissatisfaction and body image concerns, while considering the pivotal role of their partners’ commentary. Positive aspects of body image, such as body compassion, may be also be considered within therapies for eating disorders or supportive therapies for obese and overweight people [58]. Promoting more supportive communication within a couple might be of critical importance to foster women’s couple satisfaction. The role of communication within the relationship should be considered whether in counseling or in personal and couple therapy.

## Figures and Tables

**Table 1:** Means (*SDs*) of women’s body dissatisfaction, body compassion, and quality of couple relationship by experimental condition.

Experimental Condition	BSQ	BCS (Defusion)	BCS (Common Humanity)	BCS (Acceptance)	BCS (Tot)	SD	PD	PPR	FAR	*n*
Positive comment	2.36 (0.80)	3.70 (0.85)	2.78 (1.08)	3.57 (1.06)	3.31 (0.57)	3.21 (0.58)	2.59 (0.75)	3.03 (0.80)	0.57 (0.70)	36
Negative comment	2.49 (0.93)	3.68 (0.93)	2.59 (1.01)	3.13 (1.05)	3.11 (0.72)	3.18 (0.69)	2.70 (0.66)	3.25 (0.69)	1.03 (0.80)	38
Control	2.35 (0.78)	3.90 (0.75)	2.50 (0.98)	3.34 (0.94)	3.23 (0.60)	3.19 (0.66)	2.59 (0.74)	3.16 (0.76)	1.00 (0.81)	36

Note: BSQ-14, Body Shape Questionnaire-14; BCS, Body Compassion Scale; SD, Self-disclosure; PD, Partner-disclosure; PPR, Perceived Partner Responsiveness; FAR, Fears of being Abandoned and Rejected.

**Table 2:** Means (*SDs*) of men’s body dissatisfaction, body compassion and, quality of couple relationship by experimental condition.

Experimental Condition	MBAS (Muscularity)	MBAS (Low Body Fat)	MBAS (Height)	MBAS (Tot)	BCS (Defusion)	BCS (Common Humanity)	BCS (Acceptance)	BCS (Tot)	SD	PD	PPR	RC	*n*
Positive comment	2.69 (0.87)	2.60 (1.19)	2.49 (1.49)	2.59 (0.70)	4.25 (0.74)	2.00 (0.86)	3.90 (0.89)	3.29 (0.43)	2.58 (0.78)	2.54 (0.67)	2.96 (0.83)	1.98 (1.05)	35
Negative comment	2.57 (0.81)	3.41 (1.37)	2.85 (1.49)	2.95 (0.66)	4.04 (0.70)	1.91 (0.82)	3.56 (1.00)	3.10 (0.40)	2.66 (0.74)	2.63 (0.70)	3.14 (0.62)	2.20 (1.06)	33
Control	2.61 (0.88)	2.79 (1.01)	2.17 (1.28)	2.57 (0.65)	4.37 (0.64)	2.05 (1.11)	3.93 (0.84)	3.36 (0.52)	2.59 (0.88)	2.64 (0.55)	3.03 (0.76)	2.22 (1.00)	33

Note: MBAS, Male Body Attitudes Scale; BCS, Body Compassion Scale; SD, Self-disclosure; PD, Partner-disclosure; PPR, Perceived Partner Responsiveness; RC, Relational Communication.

## Data Availability

The data that support the findings of this study are available upon request from the corresponding author. The data are not publicly available due to their containing information that could compromise the privacy of the research participants. However, should anyone need to access a specific part of the datasets, we will do our best to comply with their request.

## Appendix A

*Positive comment condition vignette (men version)*

Summers’s coming, and you and your partner have booked a week’s vacation to a sea resort. One afternoon, you decide to go shopping together since you need a new swimsuit. You enter a shop and, after having taken a look around, you see a Speedos that you like, so you decide to try it on.

You head to the dressing room and ask your partner to come with you. You wear the swimsuit and pull back the curtain to show it to your partner.

She looks at you and says, “Wow! You look great! You’ll cut a fine figure on the beach”.

*Positive comment condition vignette (women version)*

Summers’s coming, and you and your partner have booked a week’s vacation to a sea resort. One afternoon, you decide to go shopping together since you need a new swimsuit. You enter a shop and, after having taken a look around, you see a bikini that you like, so you decide to try it on.

You head to the dressing room and ask your partner to come with you. You wear the swimsuit and pull back the curtain to show it to your partner.

He looks at you and says, “Wow! You look great! You’ll cut a fine figure on the beach”.

*Negative comment condition vignette (men version)*

Summers’s coming, and you and your partner have booked a week’s vacation to a sea resort. One afternoon, you decide to go shopping together since you need a new swimsuit. You enter a shop and, after having taken a look around, you see a Speedos that you like, so you decide to try it on.

You head to the dressing room and ask your partner to come with you. You wear the swimsuit and pull back the curtain to show it to your partner.

She looks at you and says, “Mhm…You aren’t in such a great shape. You won’t cut a fine figure on the beach”.

*Negative comment condition vignette (women version)*

Summers’s coming, and you and your partner have booked a week’s vacation to a sea resort. One afternoon, you decide to go shopping together since you need a new swimsuit. You enter a shop and, after having taken a look around, you see a bikini that you like, so you decide to try it on.

You head to the dressing room and ask your partner to take you. You wear the swimsuit and pull back the curtain to show it to your partner.

He looks at you and says, “Mhm...You aren’t in such a great shape. You won’t cut a fine figure on the beach”.
